# Lacrimal Sac and Nasolacrimal Duct Tumors Mimicking Chronic Inflammation: A Systematic Review

**DOI:** 10.3390/medicina62010142

**Published:** 2026-01-10

**Authors:** Alina Elisabeta Anglitoiu, Karina Cristina Marin, Felix Bratosin, Robert Avramut, Ovidiu Boruga

**Affiliations:** 1 Department of Otorhinolaryngology, Victor Babes University of Medicine and Pharmacy, 300041 Timisoara, Romania; alinaanglitoiu@gmail.com; 2 Clinic of Otorhinolaryngology, Pius Brinzeu Timis County Emergency Hospital, 300723 Timisoara, Romania; 3 Department of Infectious Diseases, Faculty of Medicine, Victor Babes University of Medicine and Pharmacy, 300041 Timisoara, Romania; felix.bratosin@umft.ro; 4 Faculty of Medicine, Doctoral School, Victor Babes University of Medicine and Pharmacy, 300041 Timisoara, Romania; robert.avramut@umft.ro; 5 Department of Oral and Maxillofacial Surgery, Faculty of Dentistry, Victor Babes University of Medicine and Pharmacy, 300041 Timisoara, Romania; 6 Discipline of Ophthalmology, Faculty of Medicine, Victor Babes University of Medicine and Pharmacy, 300041 Timisoara, Romania; ovidiu.boruga@umft.ro

**Keywords:** lacrimal sac, nasolacrimal duct obstruction, dacryocystorhinostomy, carcinoma, squamous cell, biopsy

## Abstract

*Background and Objectives:* Lacrimal sac/nasolacrimal duct (LS/NLD) tumors may present as primary acquired nasolacrimal duct obstruction (PANDO), raising debate over routine versus selective dacryocystorhinostomy (DCR) biopsy. We systematically reviewed (i) biopsy yields in routine versus selective strategies, (ii) clinical/imaging red flags for neoplasia, and (iii) outcomes of malignant LS/NLD tumors. *Materials and Methods:* Following a preregistered PRISMA 2020-compliant protocol, we searched PubMed/MEDLINE, Web of Science, and Scopus (1970–2025) for adult cohorts reporting histopathology, imaging, or oncologic outcomes in PANDO/DCR or LS/NLD tumors. Eligible designs included comparative, cohort, cross-sectional, and diagnostic accuracy studies with histology as a reference. *Results:* Across 16 cohorts, routine DCR series reported “any specific pathology” in 0–7.91% of specimens and malignant yields generally ≤0.73%. In Anderson, 7.91% of 316 patients had significant pathology and 4.43% neoplasia, with 2.53% unsuspected pre-/intra-operatively. Selective biopsy or tumor-enriched cohorts showed higher malignant burdens; pooled modern data yielded ~72.8% squamous cell carcinoma and ~21.4% lymphoma among malignancies. Imaging red flags included bone erosion (50% malignant vs. 11% benign) and infiltrative patterns (63% vs. 0%), while sac masses were present in 88% of tumors in one recent series. In LSSCC-only cohorts, contemporary multimodal therapy achieved 5-year overall survival of 87.6% and progression-free survival of 76.3%. *Conclusions:* Malignancy is rare in unselected PANDO but clinically significant when present. A tiered strategy combining bedside red flags, targeted CT/MRI, and selective biopsy appears to balance oncologic safety with resource stewardship and supports histology-directed epithelial versus lymphoma care pathways.

## 1. Introduction

Primary acquired nasolacrimal duct obstruction (PANDO) dominates adult epiphora, yet a clinically important minority of patients harbor lacrimal sac or nasolacrimal duct (LS/NLD) tumors that initially masquerade as chronic dacryocystitis or “simple” obstruction, delaying definitive oncologic care and risking orbital, sinonasal, and skull-base spread [1,2,3]. Classic clinicopathologic cohorts already underscored that a substantial fraction of LS/NLD neoplasms present with non-specific tearing and medial canthal fullness rather than a frank mass, challenging early triage at the oculoplastic–rhinology interface [1,2]. Contemporary series reiterate this diagnostic fog and quantify the price of delay—long symptom-to-diagnosis intervals, higher-stage disease, and escalated treatment intensity once tumors finally declare themselves [4].

Because LS/NLD pathology straddles two anatomies and two clinics (medial canthus–lacrimal fossa for ophthalmology; lateral nasal wall–inferior meatus for ENT), imaging has become the shared language for discerning when “dacryo” might actually be “onco.” High-resolution CT delineates sac-centered soft tissue, widening of the bony nasolacrimal duct, focal lamina papyracea remodeling/erosion, and inferior meatal extension; MRI refines characterization with enhancement patterns, marrow signal, perineural trajectories, and post-contrast interfaces—patterns repeatedly linked to LS/NLD neoplasia and actionable surgical planning [5,6]. Red-flag bedside clues—particularly a firm, non-compressible medial canthal lesion above the medial canthal tendon or hemolacria (blood-stained tears)—are uncommon, but are specific triggers that should prompt targeted cross-sectional imaging and an oncologic workflow rather than routine dacryocystorhinostomy (DCR) alone [4,7].

Histologically, epithelial malignancies predominate in the lacrimal sac, led by squamous cell carcinoma (SCC), with inverted/transitional papilloma (premalignant), adenocarcinoma, mucoepidermoid, and adenoid cystic subtypes in the mix; lymphomas (MALT, DLBCL) comprise the leading non-epithelial group and may present without systemic disease at first contact [3,8,9,10,11,12]. Molecular pathology is beginning to illuminate etiology and risk: high-risk HPV-related SCC of the lacrimal sac shows p16/Rb signatures reminiscent of sinonasal oncogenesis, and early exome/transcriptome work is mapping targetable drivers across lacrimal drainage carcinomas [13,14]. Imaging case series also highlight distinctive phenotypes (e.g., T1-bright melanoma; subtle, enhancing sac masses without obvious bone change for lymphoma), sharpening differential diagnosis and biopsy thresholds [4,5,6,12,15].

Whether to biopsy the sac routinely at DCR or selectively remains an enduring controversy. A comprehensive review aggregating 3865 routine DCR biopsies found non-specific chronic inflammation in ~94%, “specific” pathologies (including tumors, granulomatous disease, dacryoliths) in ~6%, and neoplasia in ~1.4% overall—of which many malignancies were not suspected pre-/intra-operatively [8]. Yet, prospective and institutional series argue that, in typical PANDO, the incremental management value of blanket histology is limited and cost-intensive, while selective programs that biopsy only when clinical or intra-operative appearances are atypical (e.g., fibrotic sac, fistula, bloody reflux) capture most tumors with far fewer specimens [9]. Pragmatically, a tiered pathway that couples red-flag screening with CT/MRI and selective biopsy appears to balance the very low base rate of malignancy against the very high cost of missing one [4,5,6,7,8,9].

Oncologic outcomes reinforce this triage philosophy. For sac SCC, multimodal therapy anchored in complete oncologic resection with margin control—often via combined ophthalmology–endoscopic ENT approaches—and adjuvant radiotherapy yields the best local control, while definitive radio(chemo)therapy is a reasonable alternative in unresectable disease or when exenteration is declined, with modern series reporting encouraging 2–5-year survival and locoregional control rates alongside predictable ocular toxicities [10]. Lymphomas of the lacrimal sac, in contrast, are frequently amenable to radiotherapy (with or without systemic therapy) with high local control and vision preservation when recognized early, a further argument for imaging-triggered, selective biopsy rather than indiscriminate sampling [11,12]. Rare but consequential entities—HPV-related SCC, inverted papilloma with malignant potential, primary melanoma, and even primary adenocarcinoma controlled long-term with radiotherapy—round out today’s differential and illustrate why a shared ENT–ophthalmology algorithm focusing on red flags, targeted imaging, and selective histology is both safe and efficient [13,15,16,17,18,19,20].

Therefore, the current study aims to answer the following questions: (1) What are the numerical yields of neoplasm detection in routine vs. selective DCR biopsy? (2) Which CT/MRI signs and clinical flags should trigger ENT–ophthalmology oncologic workflows? (3) What are the outcomes of malignant LS/NLD cohorts?

## 2. Materials and Methods

### 2.1. Protocol, Registration, and Reporting Standards

This review was prospectively designed according to PRISMA 2020 [21] and the Methodological Expectations of Cochrane Reviews. A protocol detailing objectives, eligibility criteria, primary and secondary outcomes, and analysis plans was preregistered on the Open Science Framework (OSF; identifier osf.io/3pcu7). Any post hoc amendments, such as expanding imaging synonyms to better capture sinonasal extension of lacrimal drainage tumors, were documented with date-stamped notes. The PRISMA 2020 checklist and flow diagram will be provided as Supplementary Material Table S1.

### 2.2. Research Question and PICO Statement

We asked, in adults evaluated for epiphora or primary acquired nasolacrimal duct obstruction (PANDO) or presenting with lacrimal sac/nasolacrimal duct (LS/NLD) lesions, what are the detection yields of routine versus selective dacryocystorhinostomy (DCR) biopsies for neoplasia, which clinical and imaging features are associated with neoplasia, and what oncologic outcomes are reported for malignant LS/NLD tumors. The population comprised adults (≥16 years) managed in ophthalmology and/or otorhinolaryngology services for PANDO, DCR, or suspected LS/NLD tumors. The interventions/exposures were routine sac biopsy at DCR, selective biopsy triggered by predefined “red flags,” and CT/MRI features suggestive of neoplasia, as well as oncologic treatments such as surgery with or without radiotherapy or chemotherapy. Comparators were routine versus selective biopsy strategies, neoplastic versus non-neoplastic pathology, alternative imaging features, and different oncologic modalities where comparable. Primary outcomes were the proportions of “any neoplasia,” “malignant neoplasia,” and “unsuspected malignancy” detected at or after DCR; secondary outcomes included diagnostic accuracy of imaging and clinical red flags against histology, time from symptom onset to diagnosis, local control and survival, exenteration rates, complications, and covariates associated with malignancy.

### 2.3. Eligibility Criteria

We included randomized or non-randomized comparative studies, prospective or retrospective cohorts, cross-sectional clinico-pathologic series, and diagnostic accuracy studies that enrolled adult humans and reported at least one prespecified outcome relevant to biopsy yields, imaging red flags, or oncologic outcomes in LS/NLD disease. We excluded case reports and case series with fewer than ten patients, conference abstracts without extractable data, narrative reviews, editorials, pediatric-only cohorts, animal studies, and reports lacking a histopathology reference standard for accuracy analyses. For overlapping cohorts from the same center and time period, we retained the most complete or most recent report and used companion papers only to supplement missing variables without double counting.

### 2.4. Information Sources

The primary databases were PubMed/MEDLINE, Web of Science Core Collection (SCI-Expanded, SSCI, ESCI), and Scopus. To maximize completeness, we conducted backward and forward citation chasing for all included records, hand-searched key journals in ophthalmology, oculoplastic surgery, rhinology, head-and-neck oncology, and neuroradiology, and screened ClinicalTrials.gov to identify completed studies with published data. No language restrictions were imposed at the database level; non-English full texts were translated where feasible.

### 2.5. Search Strategy

All searches were PRESS-checked and executed most recently on 1 August 2025, limiting by publication date but not language and excluding animal-only records. In PubMed/MEDLINE, we used the following structured string written into a single query: “(‘Lacrimal Sac’[Mesh] OR ‘Nasolacrimal Duct’[Mesh] OR lacrimal sac[tiab] OR nasolacrimal duct[tiab] OR NLD[tiab] OR LS/NLD[tiab] OR dacryocyst*[tiab] OR ‘primary acquired nasolacrimal duct obstruction’[tiab] OR PANDO[tiab]) AND ((tumor*[tiab] OR tumour*[tiab] OR neoplasm*[tiab] OR carcinoma[tiab] OR lymphoma[tiab] OR papilloma[tiab] OR melanoma[tiab] OR ‘squamous cell’[tiab] OR adenocarcinoma[tiab]) OR ((dacryocystorhinostomy[tiab] OR DCR[tiab]) AND (biopsy[tiab] OR histolog*[tiab] OR patholog*[tiab]))) AND (CT[tiab] OR ‘computed tomography’[tiab] OR MRI[tiab] OR ‘magnetic resonance’[tiab] OR imaging[tiab] OR ‘red flag*’[tiab] OR hemolacria[tiab] OR ‘blood-stained tear*’[tiab] OR ‘medial canthal’[tiab] OR mass[tiab]) NOT (animals[mh] NOT humans[mh]) AND (‘1 January 1970′[Date—Publication]: ‘23 September 2025′[Date—Publication]).”

In Web of Science Core Collection, we used a topic query spanning title, abstract, and keywords as follows: “TS = ((‘lacrimal sac’ OR ‘nasolacrimal duct’ OR dacryocyst* OR PANDO OR ‘primary acquired nasolacrimal duct obstruction’) AND (tumor* OR tumour* OR neoplasm* OR carcinoma OR lymphoma OR papilloma OR melanoma OR ‘squamous cell’ OR adenocarcinoma OR ((DCR OR dacryocystorhinostomy) NEAR/3 (biopsy OR histolog* OR patholog*))) AND (CT OR ‘computed tomography’ OR MRI OR ‘magnetic resonance’ OR imaging OR ‘red flag*’ OR hemolacria OR ‘blood-stained’)),” limited to 1970–2025 and document types with full data. In Scopus, we searched titles, abstracts, and keywords with the following: “TITLE-ABS-KEY ((‘lacrimal sac’ OR ‘nasolacrimal duct’ OR dacryocyst* OR PANDO OR ‘primary acquired nasolacrimal duct obstruction’) AND (tumor* OR tumour* OR neoplasm* OR carcinoma OR lymphoma OR papilloma OR melanoma OR ‘squamous AND cell’ OR adenocarcinoma OR ((DCR OR dacryocystorhinostomy) AND (biopsy OR histolog* OR patholog*))) AND (CT OR ‘computed AND tomography’ OR MRI OR ‘magnetic AND resonance’ OR imaging OR ‘red AND flag*’ OR hemolacria OR ‘blood-stained’)) AND (PUBYEAR > 1969 AND PUBYEAR < 2026) AND DOCTYPE(ar).” We iteratively tested synonyms and hyphenation variants (for example, “dacryocyst*” to encompass DCR/DCT variants) to maintain sensitivity while limiting noise.

### 2.6. Study Selection and PRISMA Flow

**Figure 1:**
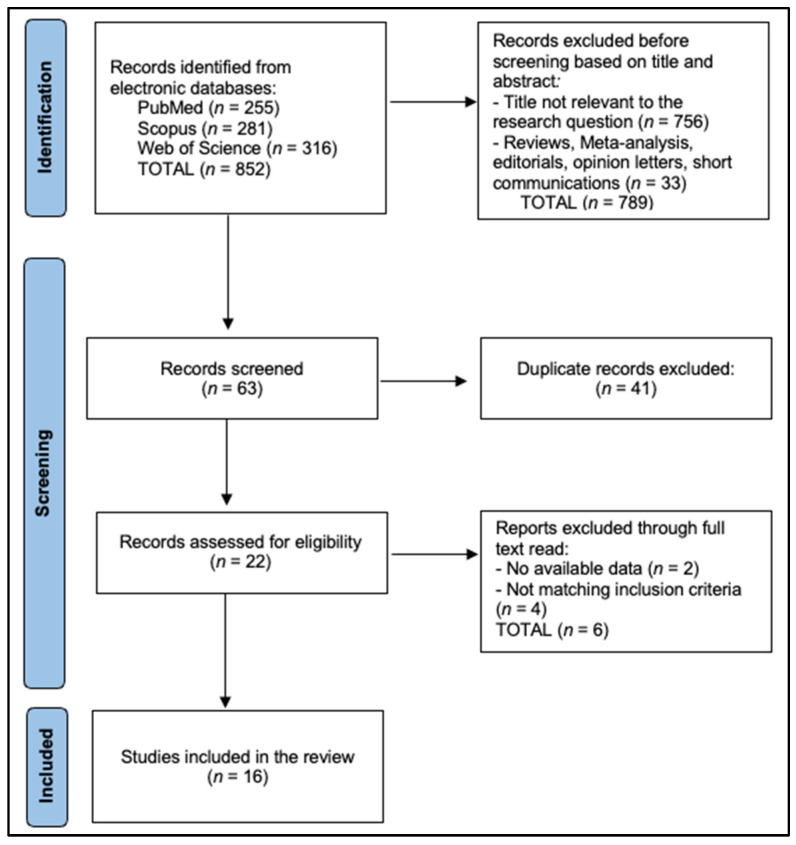
PRISMA Flowchart Diagram.

All records were exported, de-duplicated in EndNote X20 using DOI, author, title, and journal fields with manual review of near-duplicates, and imported to Rayyan QCRI for independent, blinded title/abstract screening by two reviewers. Full texts were obtained for potentially eligible studies and assessed against inclusion criteria with prespecified exclusion reasons recorded (for example, pediatric-only cohorts, absence of histology reference standard for imaging accuracy, failure to report denominators for yield outcomes, or overlap with a more complete cohort). Disagreements were resolved through discussion or third-reviewer arbitration (Figure 1).

### 2.7. Data Items and Extraction Procedures

We extracted study metadata (year, country, setting, design), cohort definitions (PANDO DCR program versus tumor registry; routine versus selective biopsy policy and criteria), sample size and demographics, clinical red flags (hemolacria, firm or non-compressible mass above the medial canthal tendon, fistula, cranial neuropathies), imaging protocols and features (CT kernel and slice thickness, MRI sequences and contrast timing, sac-centered soft tissue, nasolacrimal duct widening, bony remodeling or erosion, sinonasal extension, perineural spread), surgical approaches (external versus endoscopic DCR, combined ENT–ophthalmology management), histopathology categories (benign, premalignant, malignant subtypes), primary outcomes (proportions for any neoplasia, malignant neoplasia, and unsuspected malignancy), diagnostic accuracy data (2 × 2 tables for red flags and imaging versus histology), time intervals from symptom onset to diagnosis, oncologic treatments and outcomes (local control, survival, exenteration, complications), and covariates (prior DCR, dacryoliths, granulomatous disease).

For tumor cohorts, we additionally extracted (when reported) the operative corridor (external/open, endoscopic endonasal, or combined), reconstruction/defect management, and adjuvant therapy details (radiotherapy fields/doses and systemic regimens for lymphoma).

### 2.8. Risk of Bias Assessment

Two reviewers independently appraised study quality using design-specific tools with consensus adjudication. Diagnostic accuracy studies employing imaging or clinical red flags against histopathology were assessed with QUADAS-2 across patient selection, index test, reference standard, and flow/timing, including applicability concerns. Non-randomized comparative cohorts or before–after designs addressing oncologic outcomes or strategy comparisons were assessed with ROBINS-I. Observational cohorts and cross-sectional clinico-pathologic series reporting biopsy yields were assessed with the Newcastle–Ottawa Scale or the JBI checklist for case series with at least ten patients. Domain-level judgments were summarized narratively and graphically.

## 3. Results

Across 16 cohorts [22,23,24,25,26,27,28,29,30,31,32,33,34,35,36,37,38] spanning 1986–2024, routine DCR biopsy series reported “any neoplasia/specific pathology” between 0 and 7.91% and malignant yields typically ≤0.73%. Specifically, Anderson (*n* = 316) observed 7.91% significant pathology and 4.43% malignant, with 2.53% unsuspected pre-/intra-op [23]; Bernardini (302 specimens) found 3.31% positives overall, with 8/10 positive cases having an abnormal-appearing sac [22]; Alkatan (498 specimens) reported 3.43% unsuspected benign/specific pathology, 0% malignant, and only 0.6% pre-op suspicion [28]; Makselis (*n* = 275 ANLDO) identified tumors in 1.09% with 0.73% malignant and 0.7% palpable mass at presentation [29]; Merkonidis (193 DCRs) found 1.55% “specific” pathology (two sarcoid, one papilloma) and 0.52% neoplastic, mean age 64 years, 65.9% female [25]; Altan-Yaycioglu (*n* = 205) recorded 0.49% malignant [26]; and Salour (471 specimens) detected 0.42% lymphoma, with 0.21% unsuspected malignant [27]. Tumor registry or malignant-only cohorts expectedly had higher malignant proportions: Kuo (*n* = 65 lesions) reported 29.2% malignant with imaging red flags (bone erosion 50% vs. 11% benign; infiltrative pattern 63% vs. 0%) [33], while Wakasaki (*n* = 25 tumors) had 88% malignant with 88% presenting with a sac mass [35], and Song analyzed LSSCC exclusively (*n* = 69) [34]. ENT co-management was common in endoscopic series and multidisciplinary teams predominated in overt tumor cohorts [25,30,35], as described in Table 1.

Orbital signs were uncommon in routine PANDO/DCR cohorts (typically not reported), but they were documented in tumor-enriched series. In a tertiary lacrimal sac lesion cohort comparing benign versus malignant disease, proptosis occurred in 20% (2/10) of primary malignant cases and extraocular motility limitation in 10% (1/10), supporting orbital mass effect and/or early extension as clinically meaningful red flags. In a delayed/misdiagnosed tumor series, proptosis was present in 1/11 (9.1%), and one patient with adenoid cystic carcinoma required orbital exenteration with skull-base surgery to achieve optimal surgical margins.

Malignant-focused cohorts underscore delayed diagnosis, aggressive imaging features, and the value of multimodal therapy. In Kuo, malignant lesions more frequently showed bone erosion (50% vs. 11% benign) and an infiltrative pattern (63% vs. 0%), with treatment skewed to surgery (100%) plus adjuvant RT (42.1%) or CRT (47.4%); recurrence, metastasis, and mortality reached 42%, 47%, and 53%, respectively [33]. Wakasaki documented a mean diagnostic delay of 14.7 months (median 8; range 1–96), predominant surgical management for epithelial tumors (93.3%), selective use of heavy-ion RT and immune checkpoint inhibition, and local control in all but one case; histologies were SCC (*n* = 6), lymphoma (*n* = 10), adenoid cystic (*n* = 2), sebaceous (*n* = 2), and MEC (*n* = 1) [35]. In LSSCC-only series, contemporary strategies (surgery + adjuvant vs. definitive radio[chemo]therapy) achieved 5-year OS 87.6% and PFS 76.3%, with nodal positivity an adverse factor [34]. A selective biopsy policy captured malignant and specific inflammatory entities while limiting biopsies; 5-year survival was 87% with significant pathology vs. 96% without [32]. Routine DCR series contribute malignant identifiers but often lack detailed outcome denominators (e.g., Makselis: adenoid cystic *n* = 1; small B-cell lymphoma *n* = 1; Salour: lymphoma *n* = 2) [27,29], as seen in Table 2.

Where specified, epithelial malignancies were managed with oncologic resection using an open (external) approach, endoscopic endonasal approach, or combined corridor, selected according to anatomic extent (sac-confined vs. sinonasal/orbital/skull-base extension). In a malignant lesion cohort, all malignant cases underwent surgical resection, with adjuvant radiotherapy in eight cases and concurrent chemoradiotherapy in nine cases. In delayed/misdiagnosed tumors, postoperative radiotherapy/chemoradiotherapy was frequently used, and extensive disease occasionally required orbit-skull-base surgery for margin control.

**Figure 2:**
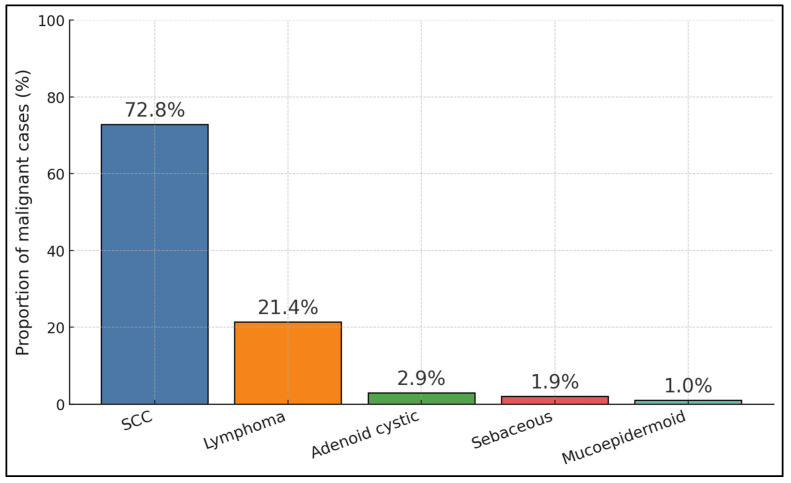
Malignant histology composition across tumor cohorts (percentage).

Pooled across modern tumor-enriched datasets, SCC comprised ~72.8% of malignancies (75/103), lymphoma ~21.4% (22/103), with adenoid cystic ~2.9% (3/103), sebaceous ~1.9% (2/103), and MEC ~1.0% (1/103), reflecting an epithelial-dominant profile with a meaningful lymphoma minority [29,34,35,36]. Clinically, this split supports two parallel care pathways: oncologic resection plus adjuvant RT/CRT for SCC-predominant disease (often via combined ophthalmology–endoscopic ENT approaches), and hematologic staging with RT/chemo for lacrimal sac lymphomas—both optimized within joint ENT–ophthalmology workflows [29,34,35,36], as presented in Figure 2.

**Figure 3:**
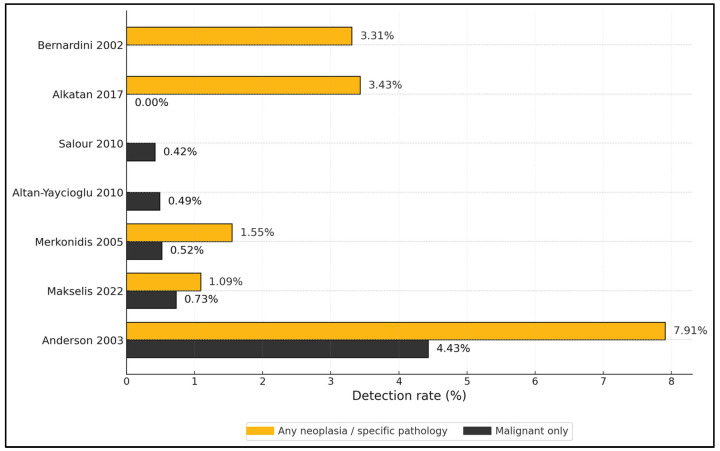
Detection rates in routine DCR biopsy series [22,23,25,26,27,28,29].

Routine, unselected PANDO cohorts show low malignant yields (≈0–0.73%) alongside modest “any specific pathology” rates: Anderson reported 7.91% significant pathology and 4.43% malignant among 316 patients [23]; Bernardini observed 3.31% positives in 302 specimens [22]; Alkatan found 3.43% unsuspected benign/specific pathology and 0% malignant in 498 specimens [28]; Makselis reported 1.09% tumors with 0.73% malignant in 275 ANLDO cases [29]; Merkonidis identified 1.55% specific pathology (0.52% neoplastic) in 193 DCRs [25]; Altan-Yaycioglu and Salour recorded 0.49% and 0.42% malignant, respectively [26,27]. These numerics reinforce a selective biopsy strategy guided by red flags and imaging rather than blanket histology in typical PANDO presentations [22,23,25,26,27,28,29], as described in Figure 3.

Stepwise algorithm beginning with bedside screening for red flags (mass above medial canthal tendon, hemolacria/blood-stained reflux, firm non-compressible lesion, recurrent “dacryocystitis” despite treatment, cranial neuropathies, proptosis/EOM limitation, suspicious intranasal findings). If red flags are present, proceed to contrast CT and/or MRI to assess sac-centered mass, nasolacrimal duct widening, infiltrative margins, bony remodeling/erosion, sinonasal/orbital extension, and perineural spread. Tissue diagnosis is obtained via targeted biopsy (or intraoperative biopsy if proceeding to surgery). Management then diverges to epithelial oncologic resection ± adjuvant radiotherapy/chemoradiotherapy versus lymphoma work-up with hematologic staging and organ-preserving radiotherapy/systemic therapy, as indicated.

**Figure 4:**
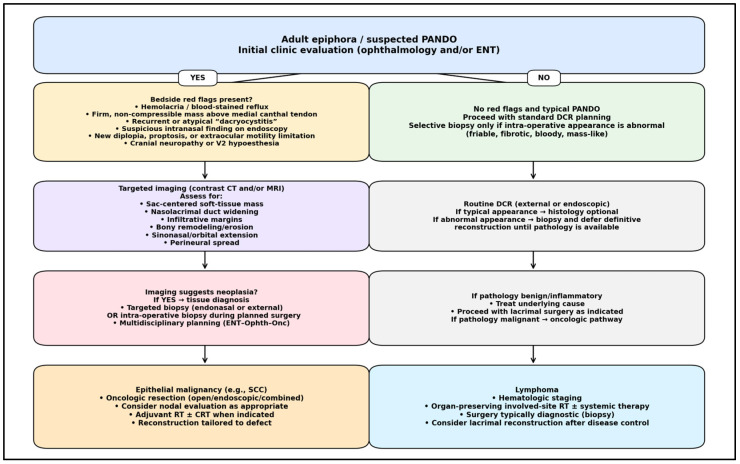
Tiered clinico-radiological work-up for adult epiphora/PANDO to identify lacrimal sac/nasolacrimal duct (LS/NLD) neoplasia.

Figure 4 summarizes the core synthesis of this review: most typical PANDO presentations can proceed with standard dacryocystorhinostomy, whereas a defined set of bedside red flags should trigger targeted cross-sectional imaging and selective tissue diagnosis. This tiered approach operationalizes how radiologic indicators of neoplasia (infiltrative sac-centered mass, nasolacrimal duct widening with bony change, and extension patterns) shift care from routine lacrimal surgery to an oncologic pathway, with histology determining epithelial versus lymphoma-specific treatment.

## 4. Discussion

### 4.1. Summary of Evidence

Our findings that epithelial malignancies, particularly squamous cell carcinoma (SCC), predominate among lacrimal sac/nasolacrimal duct (LS/NLD) tumors while lymphomas represent a substantial minority are consistent with historical oncologic series focused on malignant lacrimal sac disease. In a frequently cited institutional experience, more than half of lacrimal sac tumors were malignant overall and most primary lesions were epithelial, reinforcing the dual-track clinical logic we propose—definitive resection plus adjuvant radiotherapy for epithelial cancers versus hemato-oncologic staging with site-directed radiotherapy for lymphomas [39]. The malignant proportion in that series is understandably higher than in our unselected PANDO cohorts, but the histologic hierarchy overlaps and supports our recommendation to keep SCC and lymphoma at the top of the differential once red flags emerge.

Imaging cues that we identified—sac-centered soft tissue with nasolacrimal canal remodeling or erosion, infiltrative margins on CT/MRI, and sinonasal/orbital extension—map closely onto contemporary surgical reports where cross-sectional suspicion directly shapes the operative corridor. For example, an endoscopy-assisted modified Weber–Ferguson approach achieved durable control in primary lacrimal sac tumors with extra-sac extension, and the authors emphasized uneven enhancement and complex regional spread as preoperative red flags that justified combined access for margin control and cosmesis—principles that mirror our escalation pathway from “routine DCR” to “oncologic resection” once imaging turns atypical [40]. A 2024 literature review of 316 patients likewise concluded that minimally invasive endoscopic routes—used alone or combined—are safe and oncologically sound when imaging indicates lacrimal-pathway neoplasia, with SCC most prevalent and lymphomas and melanomas comprising important minorities—again echoing our pooled histology profile and team-based management model [41].

A practical barrier to cross-study synthesis we encountered is staging heterogeneity. Unlike many sinonasal sites, lacrimal sac carcinomas currently rely on Summary Stage 2018 rather than a dedicated TNM schema, which limits direct stage-matched outcome comparisons across cohorts and favors an anatomic-extent vocabulary (sac-confined vs. sinonasal, orbital, bony, or nodal spread) for reproducible reporting. National registry guidance explicitly notes that Summary Stage is the only applicable system for the lacrimal sac, a point that aligns with our call to standardize extent-of-disease descriptors and to integrate nodal assessment into baseline work-ups when malignancy is suspected [42]. This clarification also helps interpret why “time to diagnosis” and “stage at presentation” varied widely in the literature feeding our results—differences in reporting frameworks were at least partly to blame.

Our outcome synthesis—strong local control with complete resection plus adjuvant radiotherapy for resectable epithelial tumors, with organ-preserving definitive (chemo)radiotherapy as a rational alternative when surgery is not feasible—finds growing support in the modern head-and-neck literature applied to nasolacrimal tract SCC. Parallel to our pooled malignant cohorts, contemporary series emphasize that regional nodal status and local extent remain the principal adverse factors; they also highlight the value of multidisciplinary decision-making at the oculoplastic–rhinology–radiation oncology interface to balance oncologic margins, ocular function, and cosmetic endpoints [41]. Precision oncology case reports further underscore why tissue should be leveraged beyond H&E: HER2-amplified lacrimal sac SCC has been documented with immunohistochemical and FISH confirmation, expanding the roster of potentially actionable alterations and supporting our recommendation for comprehensive immunophenotyping/molecular profiling in selected patients [43].

Lacrimal sac lymphomas in our dataset behaved distinctly from epithelial cancers, and this separation is congruent with the ocular adnexal lymphoma literature, where modest-dose radiotherapy (≈20–25 Gy in 10–15 fractions with lens shielding as indicated) yields excellent local control with limited late toxicity in stage I–II disease. This evidence base supports our algorithmic pivot—from “DCR-centric” workflows to systemic staging and site-directed radiotherapy—once histology reveals lymphoma; it also justifies keeping lymphoma in the differential when imaging shows enhancing sac masses without bone change and when clinical presentation lacks aggressive features [44]. Such alignment between our pooled outcomes and external data strengthens confidence that a selective biopsy approach, triggered by red flags and imaging rather than routine blanket sampling, will still capture treatable lymphomas early without over-biopsying typical PANDO.

From a practical standpoint, orbital involvement should be suspected when clinical findings (proptosis, diplopia, reduced extraocular motility, globe displacement, V2 hypoesthesia, or new visual symptoms) align with CT/MRI evidence of lamina papyracea erosion, post-septal fat stranding, extra-conal soft-tissue extension, or perineural spread. When these features are present, management typically escalates from “DCR-centric” care to an oncologic workflow: cross-sectional staging (contrast CT and/or MRI), multidisciplinary planning (ophthalmology–ENT–oncology), and tissue diagnosis via targeted biopsy. In extensive epithelial disease where negative margins cannot otherwise be achieved, orbital-sacrificing surgery may be required; this is illustrated by an adenoid cystic carcinoma case that underwent orbital exenteration with skull-base surgery for margin optimization.

Across modern practice, epithelial tumors of the lacrimal sac/nasolacrimal duct are typically treated with en bloc oncologic resection aiming for negative margins. Surgical access is individualized: (i) open external corridors (e.g., medial canthal/external approaches, lateral rhinotomy/Weber–Ferguson variants) are favored when broad exposure is required for bony resection, skin/soft-tissue involvement, or complex reconstruction; (ii) endoscopic endonasal approaches support sinonasal extension management, inferior meatal/NLD disease, and margin assessment under direct endoscopic visualization; and (iii) combined open–endoscopic corridors are often used when both lacrimal fossa and sinonasal compartments must be addressed. Reconstruction is dictated by defect burden and may include medial canthal soft-tissue repair, local flap closure for skin/soft-tissue deficits, lining restoration when sinonasal mucosa is resected, and selective bony/orbital wall reconstruction when stability or protection is compromised; orbital exenteration is reserved for extensive post-septal involvement when margins are otherwise unattainable.

In contrast, lacrimal drainage lymphomas are primarily managed with hematologic staging and site-directed radiotherapy (often organ-preserving), with systemic therapy guided by histologic subtype. Indolent ocular adnexal lymphomas are commonly treated with involved-site radiotherapy (often in the ~20–25 Gy range), while aggressive subtypes such as diffuse large B-cell lymphoma typically require systemic immunochemotherapy (e.g., rituximab-based regimens) with radiotherapy considered according to stage, response, and multidisciplinary judgment. In this framework, surgery is typically diagnostic (biopsy/excision for tissue) rather than definitive therapy for lymphoma.

Finally, our selective strategy also safeguards against overlooking premalignant lacrimal-pathway lesions such as inverted (Schneiderian) papilloma, which may masquerade as chronic dacryocystitis yet carry recurrence and malignant-transformation risks. Case-based reviews document that these tumors, although rare in the lacrimal sac/NLD, can be locally aggressive and benefit from complete excision with vigilant follow-up; imaging signs like the cerebriform pattern and, in selected cases, FDG-PET/CT uptake can help define extent, even if specificity for transformation remains imperfect [45,46]. Given our pooled low malignant yield in routine DCR specimens but meaningful consequences of missed neoplasia, a tiered pathway—red-flag screening, targeted CT/MRI, and selective histology—appears to balance efficiency with oncologic safety across SCC, lymphoma, and inverted papilloma phenotypes consistent with present-day literature.

### 4.2. Limitations

Heterogeneity in study designs, denominators, and pathology reporting constrained quantitative synthesis; notably, “significant pathology” variably bundled neoplasia with inflammatory/systemic entities. Staging was inconsistently defined (often using Summary Stage rather than a TNM equivalent), impeding stage-matched comparisons. Imaging protocols differed across centers (CT kernel, MRI sequences, slice thickness), limiting pooled diagnostic accuracy estimates. Many routine DCR series lacked granular outcomes (nodal status, margin control, toxicity), and several tumor-enriched cohorts were small, single-institution, and retrospective, introducing selection and referral biases. Finally, time-to-diagnosis metrics and red-flag definitions were non-standardized, which may over- or underestimate the yield of selective biopsy.

## 5. Conclusions

Routine histology during DCR in typical PANDO uncovers few malignancies, whereas a selective strategy keyed to bedside red flags and corroborative CT/MRI captures the clinically meaningful minority with cancer while minimizing unnecessary specimens. Imaging hallmarks (sac-centered soft tissue with bony remodeling/erosion or infiltrative margins) should cue multidisciplinary planning. Histology is epithelial-dominant (SCC) with a substantial lymphoma minority, justifying divergent, pathway-specific treatments. Standardizing extent-of-disease reporting, imaging protocols, and selective biopsy criteria—and prospectively recording nodal evaluation, margins, adjuvant therapy, toxicity, and patient-reported outcomes—should refine triage and improve external validity.

## Figures and Tables

**Table 1:** Included studies and baseline characteristics.

Study (Year)	Country/Setting	Cohort Focus/Strategy	N (pts)	Female (%)	Mean Age (y)	“Suspicious” Sac or Mass at Presentation	Any Neoplasia (%)	Malignant (%)	Unsuspected Malignant (%)	ENT Co-Management/Endoscopic DCR
Bernardini et al., 2002 [22]	Ophthalmology (U.S., single center)	Routine lacrimal sac biopsy during DCR	258 pts/302 specimens	65.8	NR	Abnormal sac in 8/10 positive cases	10/302 (3.31)	NR	NR	Mostly external DCR
Anderson et al., 2003 [23]	Ophthalmic Plast Reconstr Surg (U.S., Emory)	Routine pathology review of DCR specimens	316	NR	NR	Reviewed for “significant” pathology	25/316 with significant (7.9) *	14/316 neoplasms (4.43)	8/316 (2.53)	Ophthalmology; pathology lab series
Lee-Wing & Ashenhurst, 2001 [24]	Ophthalmology (Canada)	PANDO clinicopathology at DCR	166	NR	NR	NR	0	0	0	Ophthalmology
Merkonidis et al., 2005 [25]	Br J Ophthalmol (UK)	Prospective, routine wall biopsy at endoscopic DCR	164 pts/193 DCRs	65.9	64 (mean)	Inquire/inspect; select biopsy if suspicious	3/193 “specific” (1.55) **	0.52 (1 papilloma)	~1.0 (2 unsuspected systemic)	ENT + Ophth (endoscopic DCR)
Altan-Yaycioglu et al., 2010 [26]	Orbit (Turkey)	PANDO clinicopathology	205	62.4	69 (mean)	NR	NR	1/205 (0.49)	NR	Ophthalmology
Salour et al., 2010 [27]	Orbit (Iran)	Routine lacrimal sac histology during DCR	471 specimens	NR	NR	2 grossly abnormal sacs	NR	2/471 lymphoma (0.42)	1/471 (0.21)	Ophthalmology
Alkatan et al., 2017 [28]	Can J Ophthalmol (Saudi Arabia)	Routine histopathology of DCR/DCT specimens	459 pts/498 specimens	70.8	51.6 ± 17.8	0.6% pre-op suspected	17/495 unsuspected benign (3.43)	0	0	Ophthalmology
Makselis et al., 2022 [29]	BMC Ophthalmology (Lithuania)	275 ANLDO with routine lacrimal sac biopsy	275	NR	NR	0.7% palpable mass	3/275 tumors (1.09)	2/275 (0.73)	NR	Ophthalmology
Banks et al., 2020 [30]	Laryngoscope (U.S., Mass Eye and Ear)	Endoscopic DCR; role of routine sac biopsy	NR (retrospective)	NR	NR	ENT endoscopic service	NR	NR	NR	ENT lead
Eldsoky et al., 2021 [31]	Pan Arab J Ophthalmol (Egypt)	Predictive value of routine sac biopsy in endoscopic DCR	50	NR	NR	ENT endoscopic	Specific pathology yield reported	NR	NR	ENT lead
Heindl et al., 2010 [32]	Ophthalmologe (Germany)	Selective biopsies; malignant prevalence in all DCRs	~500 DCR denominator	NR	NR	Biopsy if suspicious	NR	~1.4 among all DCRs	NR	Mixed
Kuo et al., 2020 [33]	Sci Rep (Taiwan)	All lacrimal sac lesions (1995–2018)	65	NR	NR	Tumor registry	100 (by design)	29.2 malignant	NR	Ophthalmology
Song et al., 2019 [34]	Head and Neck (China)	Lacrimal sac SCC only	69	NR	NR	Cancer cohort	NR	100 SCC	NR	Multidisciplinary (ENT/Opthal/Onc)
Wakasaki et al., 2023 [35]	In Vivo (Japan)	Lacrimal sac tumors (single institution)	25	44	58.7 (avg)	88% sac mass	NR	22 malignant (88%) ***	NR	Multidisciplinary
Chu et al., 2024 [36]	Diagnostics (Taiwan)	Delayed/misdiagnosed lacrimal sac tumors	NR (retrospective)	NR	NR	Focus on delays	NR	Cohort includes multiple lymphomas	NR	Multidisciplinary
Kumar et al., 2016 [37]	AJNR (India)	Imaging spectrum of lacrimal sac pathologies	38	NR	NR	Imaging-based	NR	Mix of malignant/benign in imaging	NR	Radiology/ENT/Ophth
Linberg & McCormick, 1986 [38]	Ophthalmology (U.S.)	Excisional NLD biopsy technique; early series	14	NR	NR	Technique feasibility	NR	NR	NR	Ophthalmology

* “Significant pathology” (Anderson) includes neoplasms and specific inflammatory/systemic diseases; neoplasms themselves were 14/316, with 8/316 unsuspected pre-/intra-op. ** Merkonidis reported 3/193 “specific” pathologies (2 sarcoid; 1 papilloma); no unsuspected malignancies; mean age 64 y; 108/164 (65.9%) female. *** Wakasaki histology mix among 25 cases: SCC = 6, lymphoma = 10, adenoid cystic = 2, sebaceous = 2, MEC = 1, others benign = 3; average time to diagnosis 14.7 months; ANLDO—acquired nasolacrimal duct obstruction; DCR—dacryocystorhinostomy; DCT—dacryocystectomy; ENT—ear, nose, and throat (otolaryngology); NR—not reported; PANDO—primary acquired nasolacrimal duct obstruction.

**Table 2:** Outcomes reported with malignant tumors.

Study (Year)	Imaging Hallmarks (Malignant vs. Benign, %)	Diagnostic Delay	Treatment Composition	Nodal Status	Outcomes	Malignant Histology Details
Kuo et al., 2020 [33]	Bone erosion: 50% vs. 11% • Infiltrative pattern: 63% vs. 0%	NR	Malignant: Surgery 100%; adjuvant CRT 47.4%, RT 42.1%	NR	Recurrence 42%, metastasis 47%, mortality 53% (malignant subset)	Mixed epithelial + lymphoma cohort (counts in article)
Wakasaki et al., 2023 [35]	(qualitative; cohort-level, not % by modality)	Mean 14.7 mo; median 8 mo (range 1–96)	Epithelial tumors: Surgery 93.3%; post-op (chemo) RT in 8 cases; heavy-ion RT in 1; ICI used for 1 recurrence	NR	Local control in all but one case	SCC 6, lymphoma 10, adenoid cystic 2, sebaceous 2, mucoepidermoid 1
Song et al., 2019 [34]	NR	NR	Mixed approaches (surgery + RT vs. definitive RT/CRT; proportions not stated in abstract)	Nodal positivity present and adverse	5-yr OS 87.6%, 5-yr PFS 76.3%	SCC only (lacrimal sac)
Heindl et al., 2010 [32]	NR	—	Selective biopsy captured broad spectrum with few biopsies (policy description)	NR	5-yr survival 87% (significant pathology) vs. 96% (no significant pathology)	NHL 3, SCC 2, MEC 1, melanoma 1, oncocytoma 1, plus inflammatory/granulomatous entities
Makselis et al., 2022 [29]	NR	NR	NR	NR	NR	Adenoid cystic 1, small B-cell lymphoma 1 (malignant subtypes within total tumors)
Salour et al., 2010 [27]	NR	NR	NR	NR	NR	Lymphoma 2 (malignant IDs among routine DCR specimens)

CRT—chemoradiotherapy; ICI—immune checkpoint inhibitor; MEC—mucoepidermoid carcinoma; NR—not reported; OS—overall survival; PFS—progression-free survival; RT—radiotherapy; SCC—squamous cell carcinoma.

## Data Availability

No new data were created or analyzed in this study.

## Supplementary Materials

The following supporting information can be downloaded at: https://www.mdpi.com/article/10.3390/medicina62010142/s1, Table S1: PRISMA checklist.
